# Regulatory considerations for developing phage therapy medicinal products for the treatment of antimicrobial resistant bacterial infections

**DOI:** 10.3389/fphar.2025.1713471

**Published:** 2025-12-18

**Authors:** Ai Fukaya-Shiba, Akiko Ogata, Ryosuke Kuribayashi, Akira Sakurai, Kanako Suzuki, Shunsuke Takadama, Jihei Nishimura, Jumpei Uchiyama, Hiroki Ohge, Takamasa Takeuchi, Hideyuki Tamaki, Tetsuya Matsumoto, Kotaro Kiga, Hidetomo Iwano

**Affiliations:** 1 Office of Regulatory Science Coordination, Pharmaceuticals and Medical Devices Agency, Tokyo, Japan; 2 Office of Cellular and Tissue-based Products, Pharmaceuticals and Medical Devices Agency, Tokyo, Japan; 3 Office of Regulatory Science Research, Pharmaceuticals and Medical Devices Agency, Tokyo, Japan; 4 Office of New Drug IV, Pharmaceuticals and Medical Devices Agency, Tokyo, Japan; 5 Department of Bacteriology, Graduate School of Medicine Dentistry and Pharmaceutical Sciences, Okayama University, Okayama, Japan; 6 Department of Infectious Diseases, Hiroshima University Hospital, Hiroshima, Japan; 7 Pathogen Genomics Center, National Institute of Infectious Diseases, Japan Institute for Health Security, Tokyo, Japan; 8 Biomanufacturing Process Research Center, National Institute of Advanced Industrial Science and Technology, Ibaraki, Japan; 9 Department of Infectious Diseases, International University of Health and Welfare, Chiba, Japan; 10 Department of Drug Development, National Institute of Infectious Diseases, Japan Institute for Health Security, Tokyo, Japan; 11 Laboratory of Veterinary Biochemistry, Rakuno Gakuen University School of Veterinary Medicine, Hokkaido, Japan

**Keywords:** phage therapy, bacteriophage, antimicrobial resistance (AMR), quality considerations, non-clinical evaluation, clinical trial plan, the Cartagena Act

## Abstract

Recently, there have been growing expectations that treatment of infections with bacteriophages (phages), viruses which specifically infect bacteria, can be used as a treatment option for antimicrobial resistant bacterial infections. In Europe and the United States, in addition to phage therapy as a form of personalized medicine, development of pre-defined phage therapy medicinal products (PTMPs) is progressing, and clinical trials are underway. From October 2024 to July 2025, the Pharmaceuticals and Medical Devices Agency exchanged opinions on trends and points to consider in drug development of PTMPs used for antimicrobial resistant bacterial infections with external experts. Development of PTMPs for regulatory approval requires quality control strategies, establishment of manufacturing methods, non-clinical evaluations, and clinical trial plans based on the characteristics of the phage. In this document, based on the regulatory and development trends in Europe and the United States, the current considerations on quality, non-clinical evaluation, and clinical trial planning including the Cartagena Act in the development of PTMPs in Japan are summarized. The basic concepts presented here are intended to be applied to antimicrobial resistant bacterial infections targeted by PTMPs but can be mostly applicable to bacterial infections in general. We hope that these findings will further accelerate more active development of PTMPs towards timely patient access to innovative products.

## 1 Introduction

The Pharmaceuticals and Medical Devices Agency (PMDA) regularly exchanges with external experts to stay up to date on current trends and address relevant scientific and technological issues (PMDA, 2025a). Recent discussions focused on the recent development trends in phage therapy medicinal products (PTMPs) for the treatment of antimicrobial resistant bacterial infections. Here, we report relevant points and details to consider for the development of PTMPs in Japan.

Antimicrobial resistant bacterial infections are a serious threat, with antimicrobial resistant bacterial infections projected to be the direct cause of 1.91 million deaths per year by 2050. The cumulative number of deaths related to antimicrobial resistant bacteria from 2025 to 2050 is estimated to reach at least 169 million, including at least 39 million directly caused by such bacteria (GBD, 2021 Antimicrobial Resistance Collaborators, 2024).

Bacteriophages (hereinafter referred to as “phage”) have been used mainly in Eastern European countries as an alternative to antimicrobial drugs in the treatment of bacterial infections. Recently, however, the development of bacteriophage has progressed in Europe and the United States (U.S.) and has been increasingly drawing attention from healthcare professionals and the pharmaceutical industry as a promising alternative to antimicrobial drugs (EMA, 2023a; FDA, 2022; Global AMR R&D Hub, 2025; WHO, Regional Office for Europe, 2025). According to the World Health Organization (WHO) annual report of 2024, medicinal product candidates using phage or phage-derived enzymes account for about 10% (14 of 97) of medicinal products for treatment of bacterial infection at the clinical development stage that are not approved as a drug in any country or region (WHO, 2024). Phage-based therapy is roughly divided into “phage therapy” that is a personalized medicine using selected phages suitable for a pathogenic bacterial strain in the patient and “phage therapy medicinal products” that are standardized phage formulations with pre-defined strains, compositions and quality specifications for use across multiple patients (EMA, 2023a).

In this report, we focused on PTMPs that are standardized phage formulations, for antimicrobial resistant bacterial infections and points to consider for the future drug development of the PTMPs. Products using phage-derived enzymes such as endolysins and depolymerases are sometimes classified as PTMPs (Tan et al., 2022) but were not included in this review as they are more similar to antimicrobial peptides.

## 2 Characteristics of phages

### 2.1 Mechanism of antimicrobial action

**FIGURE 1:**
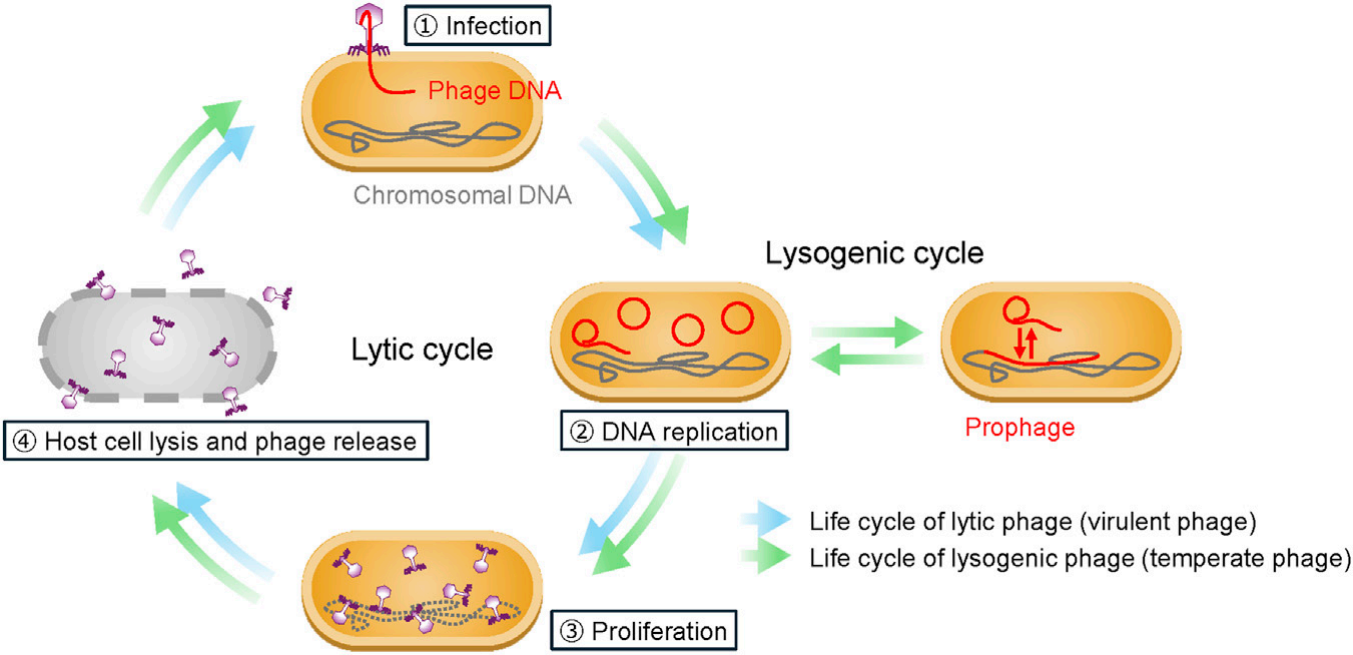
Phage life cycle. After host recognition by the phage tail, the phage genetic material is injected into the host and begins the phage proliferation cycle. After infection and phage replication, lytic phages use the host machinery to produce progeny phages. Eventually, lytic enzymes are expressed, degrading the host membrane, and thereby releasing the progeny phages to repeat the infection cycle. On the other hand, lysogenic phages can integrate their genome into the host genome without killing the host.

Phages are bacterial viruses that selectively infect their hosts to replicate. Structurally, phages are composed of a head that contains the genetic material and a tail that recognizes the bacterial host. After host recognition by the phage tail, the phage genetic material is injected into the host and begins the phage proliferation cycle. After infection and phage replication, phage-derived lytic enzymes are expressed to destroy the bacteria from the inside and release the progeny phage. PTMPs leverage the lifecycle of phage to selectively infect and kill pathogenic bacteria (Figure 1) (Iwano et al., 2019). These products are clearly differentiated from existing low-molecular antimicrobial drugs by the nature of proliferating in a bacterial body via infection with a specific bacterial species/strain after administration.

### 2.2 Lytic phages and lysogenic phages

Phages are roughly divided into lytic (sometimes “virulent”) phages and lysogenic (sometimes “temperate”) phages according to the phage lifecycle.

Lytic phages use the host machinery to produce dozens to hundreds of progeny phages. Eventually, lytic enzymes are expressed, degrading the host membrane, and thereby releasing the progeny phages to repeat the infection cycle. On the other hand, lysogenic phages can integrate their genome into the host genome without killing the host. In the host, the integrated phage can have benefits to the host, like defense against other phage infection, or be triggered to go through the lytic cycle under certain stresses. Because of these nature, lytic phages are exclusively used in the drug development of PTMPs (WHO, 2025).

### 2.3 Phage cocktail and genetically modified phages

Phage host recognition is a highly specialized process and thus phages are infectious to a limited number of strains and often highly specific to a few target strains. A phage’s host range is defined by the genetic composition of the phage, the host defense systems, the presence or absence of lysogenic phages in the bacterial host, environmental conditions, and bacterial receptor structures. These factors, amongst others, determine if a phage can recognize and replicate within a host. Additionally, genes involved in phage defense systems are known to be transferred between bacteria, partially contributing to host-range diversity.

In the case of pre-defined PTMPs, a phage’s host range may limit the overall applicability of PTMPs. As with other drugs, it is ideal that PTMPs can cover a wide range of bacterial pathogens. As the host range of phages is relatively narrow, special considerations must be taken to effectively cover a wide range of pathogenic strains. Some efforts include isolating phages with broad host ranges, using phage cocktails, phage training (Borin et al., 2021), and genetic engineering.

Phage cocktails are composed of multiple phages to cover a broad range of hosts and expected to delay the evolution of phage-resistance in bacteria. Phage training is the process of repeated coculturing of phage and bacterial host. In this process, the phage accumulates mutations that can lead to expanded host range, increased infectivity, and increased resistance to bacterial phage-resistance mechanisms. Genetically modification can expand or change a phage’s host specificity (Ando et al., 2015; Yehl et al., 2019), enhance the bactericidal ability (Krom et al., 2015; Kiga et al., 2025), improve the biofilm degradation ability (Lu and Collins, 2007), and exert a sequence-specific bactericidal effect (Bikard et al., 2014; Citorik et al., 2014; Kiga et al., 2020).

## 3 Trends in the U.S. and Europe

### 3.1 Regulatory affairs

PTMPs are classified as Biological Products by the United States Food and Drug Administration (U.S. FDA) and controlled by the Office of Vaccines Research Review under the Center for Biologics Evaluation and Research (CBER) (Plaut and Stibitz, 2021; Strathdee et al., 2023). Compassionate use is permitted in accordance with the Expanded Access Investigational New Drug under the provisions of the federal law (21CFR312.305) (FDA, 2017).

In Europe, phage therapy active substances and medicinal products is discussed in the “Biologicals” section of the European Pharmacopoeia Commission Priorities for 2023–2025 (Council of Europe, 2025). In 2023, the European Medicines Agency (EMA) published “Concept paper on the establishment of a Guideline on the development and manufacture of human medicinal products specifically designed for phage therapy” (EMA, 2023a), noting that phages differ from other medicinal products in terms of quality and therefore require special considerations. The paper outlines that future guidelines should establish phage-specific terminology, selection of starting materials, defined manufacturing process and quality control strategies, phage characterization, impurity and contamination management, development and qualification of potency assays, additional regulations for genetically modified phages, and quality requirements for products used in clinical trials.

In the same year, the guidelines for veterinary medicinal products (EMA, 2023b) were also issued and indicated requirements for marketing approval applications, such as control information, quality, safety, and efficacy as well as concomitant use with antimicrobial drugs. In July 2024, PTMPs were listed in the General Chapter (5.31) of the European Pharmacopoeia (Council of Europe, 2024), and covered, “1. Definition,” “2. Production (general provisions, bacterial cell banks, phage seed lots, production and purification, final lot, adapted product),” and “3. Labelling.” Although the chapter is not mandatory because of its non-monograph status, it outlines the minimum requirements. In June 2025, the “Regulatory considerations for therapeutic use of bacteriophages in the UK” (Medicines and Healthcare products Regulatory Agency, 2025) was issued, including the regulatory considerations applied to use of bacteriophages in the UK for human therapeutic purposes.

### 3.2 Development trend

Successful compassionate uses of phage therapy have been reported (Schooley et al., 2017; Dedrick et al., 2019). The successes prompted interest in phage therapy, which eventually led to the development of pre-defined PTMPs. However, the pre-defined PTMPs were not superior to control treatment in large-scale blinded clinical trials, with the cause suggested to be a reduced phage dose from stability issues of the product (U.S. Department of Health and Human Services, National Institutes of Health, National Library of Medicine, and National Center for Biotechnology Information, 2025; Sarker et al., 2016; European Commission, 2017; Jault et al., 2019; Leitner et al., 2021). Despite these poor results, other recent reports suggested the superiority of PTMPs (Armata Pharmaceuticals, Inc, 2024; BiomX, 2025).

Clinical trials for PTMPs development registered in Clinicaltrial.gov (U.S. Department of Health and Human Services, National Institutes of Health, National Library of Medicine, and National Center for Biotechnology Information, 2025) target *Escherichia coli*, *Pseudomonas aeruginosa*, *S. aureus*, *K. pneumoniae*, *Enterococcus*, and *Shigella* (Supplementary Table S1).

The development of PTMPs is accompanied by unique challenges. First, knowledge of the *in vivo* pharmacokinetics—specifically how phages reach and spread to infection sites—is insufficient. Secondly, there is a lack of knowledge and experience in specifying the therapeutic concentration ranges and the ideal phage host range. Lastly, clinical trials have difficulty in recruiting participants, which is a common problem with the clinical development of treatments for infectious diseases. These challenges are heightened for PTMPs, because (a) their use is likely to be considered for multidrug-resistant infections or when antibiotics are insufficient; and (b) eligible patients must carry bacterial strains covered by the host range of the PTMP. Thus, the number of eligible patients is further limited for infections caused by noncommon bacteria. Furthermore, if an epidemic strain mutates or changes, the phage strains will also likely need to be changed and thus making it difficult to establish a standardized development model.

To address these challenges, technologies such as genetic recombination and phage training can be utilized to develop phages with wider ranges or phages that can be target rapidly evolving epidemic strain. In addition, to promptly provide PTMPs to eligible patients, it is essential to develop a rapid and accurate phage sensitivity assay.

### 3.3 Development support program

In Europe, the New Drug for Bad Bugs Program, one of the Innovative Medicine Initiatives, has been instituted to construct a development platform for antimicrobial drugs and the clinical trial network (Innovative Medicines Initiative, 2025). Horizon Europe supports the research and innovation for testing safety and efficacy of treatments of antibiotic-resistant bacterial infections with phages (Horizon Europe, 2025).

In the U.S., measures implemented include establishment of Combating Antibiotic-Resistant Bacteria Biopharmaceutical Accelerator (CARB-X) (CARB-X, 2025), funding of clinical trials (U.S. Department of Health and Human Services, Administration for Strategic Preparedness and Response, 2023), and enactment of the Generating Antibiotic Incentives Now Act of 2011 (the GAIN Act) (Congress.gov, Library of Congress, 2011). In November 2024, “Combat Antibiotic Resistance Through Phage Therapy Research” was adopted as a subject to Notice of Funding Opportunity (NOFO) by the National Institute of Allergy and Infectious Diseases (NIAID) under the National Institutes of Health (NIH) (NIH, 2024).

## 4 Quality considerations

### 4.1 Quality control strategy

Development of PTMPs requires establishment of a manufacturing method that can consistently produce products with certain quality assurances and a system that can properly evaluate and control the quality of the manufactured products. Additionally, it is important to associate each quality attribute with the efficacy and safety for the quality control strategy of PTMPs. Extensive and detailed characterization needs to be performed during development. Furthermore, characterization of phages using appropriate analytical techniques (analyses on physicochemical properties, biological activity, immunochemical properties, purity, impurities, microbiological safety, and genotypic and phenotypic characteristics, etc.) will be required for the establishment of appropriate quality specifications and test methods. Acceptance criteria and process control values should be established based on data obtained from active substances and product lots used in non-clinical and clinical studies, lots demonstrating manufacturing consistency, stability study data, and other data obtained during the drug development process. Furthermore, the justification of the values must be provided. Emphasis should be placed on the selection and isolation of phage seeds and cell banks, as well as the maintenance of consistency among product lots (including genetic stability). Therefore, during the early phases of development, efforts should be focused on selection and development of a phage seed unsusceptible to gene mutations through the optimization of culture conditions wherever possible. Development of a cocktail product composed of several phage seeds requires individual characterization for each phage seed and cell bank. This characterization should elucidate the genotypes and phenotypes of each, with a focus on virulence, antimicrobial resistance, and mobile genes.

### 4.2 Phage seeds and cell banks

#### 4.2.1 Selection of phage seeds and cell banks

Phages potentially applicable for clinical use generally target virulent bacteria, with their natural hosts often being clinical isolates. As phages are generally highly specific to a few susceptible bacterial species, clinical isolates, or closely related strains, can be first choice of bacterial hosts for phage proliferation for PTMPs.

However, the use of a pathogen in the manufacturing process poses a potential biosafety issue. Furthermore, bacterial hosts have immune systems such as Clustered Regularly Interspaced Short Palindromic Repeats-Cas (CRISPR-Cas) to protect themselves from phage infection, and such systems may inhibit the proliferation of phages and thus reduce the production efficiency. Because phages prone to transduction pose a risk of genetic transfer between bacteria, phages must be tested for transduction potential and use only phages with low transduction potential. To address these issues, phages and bacteria with attributes altered by subculture or genetic engineering may be used in production.

Factors in Table 1 should be considered for the selection of a phage seed and a cell bank.

**TABLE 1:** Considerations applied to selection of phage seeds and cell banks.

Phage eligible for phage seed	Bacterial host eligible for cell bank
• Transduction potential of phage	• Bacterial virulence and ability to produce exotoxin
• Lysogeny of phage	• Gram-positive bacteria/Gram-negative bacteria
• Bacterial host range susceptible to phage	• Bacterial virulence genes and antimicrobial resistance genes
• Proliferation potential of phage	• Bacterial immune system that inhibits phage proliferation
• Presence or absence of virulence genes, antimicrobial resistance genes mobile genes, and lysogen genes	• Presence or absence of prophage integrated in the bacterial chromosome
	• Presence or absence of mobile factors such as plasmids

To characterize a phage and its bacterial host for use as a phage seed and cell bank, respectively, next-generation sequencing (NGS) and gene analysis using bioinformatics tools can be effective. Of the attributes of the cell bank listed in the above considerations, ones related to safety, such as the bacterial exotoxin genes, should be genetically deleted or adequately removed in the purification process of the active substance. Genetic engineering using homologous recombination is often effective in deleting genes. Phage training through subculture or other techniques may also provide phage strains with improved safety. If any factor raises considerable safety concern and cannot be removed, another phage or bacterial host should be selected.

There are currently no existing guidelines for the evaluation of bacterial hosts to be used for PTMPs. As host bacteria are not directly administered to humans, existing standards are not directly applicable. However, the “Points to Consider for Gut Bacterial Products Based on Microbiome Research,” (PMDA Subcommittee on Microbiome of the Science Board, 2022) a report on Live Biotherapeutic Products (LBPs) containing bacteria as the active ingredients issued by the Subcommittee on Microbiome of the Science Board, PMDA, may serve as a reference in selecting and evaluating bacterial hosts for phage production.

#### 4.2.2 Control of phage seeds and cell banks

Master cell banks (MCBs) and working cell banks (WCBs) of bacterial hosts should be established in accordance with the International Council for Harmonization of Technical Requirements for Pharmaceuticals for Human Use (ICH) Q5D guideline “Derivation and Characterisation of Cell Substrates Used for Production of Biotechnological/Biological Products” (ICH, 1997) as done for cell banks for manufacture of other biological products. Master phage seeds (MPSs) and working phage seeds (WPSs) should also be established in accordance with the ICH Q5D guideline “Derivation and Characterisation of Cell Substrates Used for Production of Biotechnological/Biological Products” (ICH, 1997).

Potential control items for phage seeds and cell banks are listed in Table 2 (Hyman, 2019; Doub, 2021) and Table 3. These control items are used to ensure that attributes investigated during selection of the phage seed and cell bank presented in Section 4.2.1 are maintained at the time of banking. Depending on attributes of the phage and cell substrates, tests may be omitted or added. In addition, the MCB, WCB, MPS, and WPS should be tested to demonstrate that the genotypes and phenotypes remain unchanged from the time of preparation and are maintained throughout the production culture period. Therefore, it is necessary to conduct tests, based on the control items listed in Tables 2, 3, on bacteria serially cultured up to the maximum number of passages expected under optimized culture conditions, as well as on the phages produced from such bacteria.

**TABLE 2:** Example of control items for MPS and WPS.

Attribute		Control item
Genotype	Whole genome sequencing	Identification of phage seed
		Presence or absence of virulence genes
		Presence or absence of antimicrobial resistance genes
		Presence or absence of lysogenicity-related genes
		Presence or absence of mobile genes (genes related to horizontal gene transfer and transformation)
Phenotype	Morphology	Transmission electron microscopy (TEM)
	Presence or absence of transduction	Transfer test of antimicrobial resistance genes
	Proliferation potential	Plaque count/liquid culture (one-step growth assay)/quantitative polymerase chain reaction (qPCR)
	Lytic potential/lysogenic potential	Genome analysis/spot assay
	Host range	Spot assay/plaque assay/liquid culture method

**TABLE 3:** Example of control items for MCB and WCB.

Attribute		Control item
Genotype	Whole genome sequencing	Identification of strains
		Presence or absence of virulence genes
		Presence or absence of antimicrobial resistance genes
		Presence or absence of mobile genetic factors (e.g., plasmid, transposon)
		Presence or absence of lysogenic phages
Phenotype	Morphology	Microscopic observations and morphology and size of colonies
	Proliferative properties	Different depending on the medium conditions
	Antimicrobial resistance	Antibiotic sensitivity test
	Motility and sporulation	Wirtz-Conklin method

Because phages are relatively prone to gene mutation, with point mutations possibly changing the phage characteristics, genetic stability must be ensured wherever possible. As with that of live vaccines, a certain level of mutations is unavoidable during the phage production. Therefore, the manufacturing method should be designed such that the inevitable mutations do not affect the efficacy and safety of the final product. To address such manufacturing challenges, it is essential to ensure the genetic stability of the bacterial host, as well as its ability to stably maintain phage genes. Therefore, in addition to selecting an appropriate strain, improving the host through genetic modification is also considered effective when necessary. Genetic stability studies should not focus on verifying the gene sequence is completely unchanged, but should confirm the efficacy and safety of the PTMP is maintained in relation to the gene sequence. If mutations are found when compared to the MPS and WPS, the identified mutations must be evaluated for the effect on clinical efficacy, safety, and product quality. For example, confirmation may involve genomic analysis or functional evaluation to ensure that the mutations do not impair bactericidal activity against the target bacteria.

### 4.3 Manufacturing method

#### 4.3.1 Manufacturing equipment

If a cell bank of the bacterium known to have virulence is used in manufacture of a PTMP, the manufacture may be likely required to be performed within a facility of the biosafety level (BSL) two or higher until inactivation of the bacterium is confirmed. It should be noted that Good Manufacturing Practice (GMP) manufacturing facilities qualified for BSL-2 or higher are very limited both in and outside Japan. In addition, if a cell bank of the genetically modified bacterial host or a phage seed of the genetically modified phage is used, they are subject to the Act on the Conservation and Sustainable Use of Biological Diversity through Regulations on the Use of Living Modified Organisms (Act No. 97 of 2003, commonly known as the Cartagena Act) (Japanese Law Translation, 2017). The competent minister is the Minister of Health, Labour and Welfare in charge of drugs, and the manufacturing facility will be required to receive “confirmation” by the competent minister responsible for containment measures for Type-2 Use of the Cartagena Act. If a phage seed of the genetically modified phage is used, its use in a clinical trial will be required to receive an Approval of Regulations on Type-1 Use of the Cartagena Act (see Section 7 “The Cartagena Act”).

#### 4.3.2 Manufacturing process

The manufacturing process is largely divided into three major processes: preparation of phage seeds and cell banks, manufacturing of the active substance, and formulation. For PTMPs, which is a drug using viruses as the active ingredients, the quality control is essentially similar to that for live attenuated viral vaccines, recombinant protein products derived from viral vectors, and other viral vector products. The following ICH guidelines on quality related to biological products should be all referred to: ICH Q5A to E “Quality of Biotechnological Products,” Q6B “Specifications,” Q8 “Pharmaceutical Development,” Q9 “Quality Risk Management,” Q10 “Pharmaceutical Quality System,” Q11 “Development and Manufacture of Drug Substances,” Q12 “Life Cycle Management.”

#### 4.3.3 Manufacture of active substance

**FIGURE 2:**
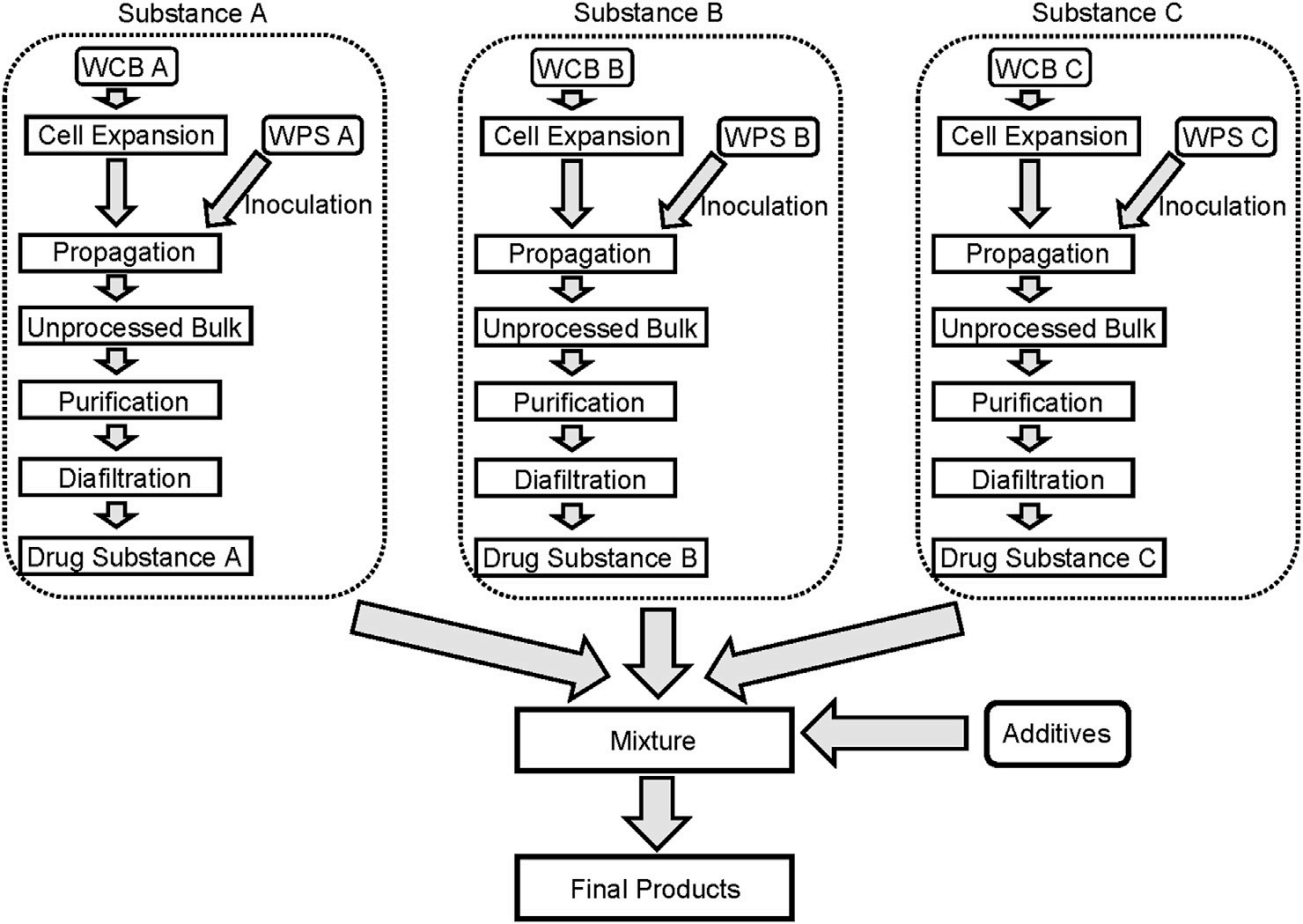
Example of manufacturing process of phage therapy medicinal product. Manufacture of the active substance mainly consists of the production culture process using the phage seed and cell bank, and the purification process. If the final product is a cocktail composed of multiple active substances, these substances are generally mixed during the formulation process, and excipients such as stabilizers are added where necessary. WCB, Working Cell Bank; WPS, Working Phage Seed.

Manufacture of the active substance mainly consists of the production culture process and the purification process. In the first process, the phage seed is incubated with large-scale culture of the bacterial host for infection, the phage released into the medium is harvested, and the crude phage bulk is subjected to filtration and chromatography for purification (Figure 2).

The purpose of the purification process is to remove host-related and process-related impurities. Because PTMPs use bacteria as the cell substrate, the freshly harvested culture medium is highly likely to contain a high concentration of endotoxins if the bacterial species is gram-negative. In some cases, the use of bacteria that produce exotoxins may be inevitable. A manufacturing method capable of removing these toxins efficiently and reliably should be established. Exotoxins need to be controlled at a level that does not affect product safety, and thus a robust removal process and quality control with strict specifications are required. Ideally, use of a non-virulent strain is desirable.

Successful cell-free synthesis of phages has been reported (Xu et al., 2025; Brooks et al., 2023; Emslander et al., 2022). Adoption of this manufacturing method would eliminate the need to use live bacteria in BSL-2 or higher facilities, and thus restrictions on the manufacturing site are expected to be relaxed. However, commercial development using this method still poses challenges. In addition, for control of impurities such as host cell proteins and nucleic acids, analytical procedures for each of the impurities need to be established to determine their individual residual amounts and thereby clarify removal of the impurities in the purification process and the residual amounts in the active substance.

For viral safety, PTMPs pose a relatively low risk compared with general biologicals manufactured using mammalian cells because the bacterial culture process cannot be contaminated with viruses potentially infectious for humans during the phage proliferation. Therefore, virus tests and viral clearance assessment in the manufacturing process are not always essential as provided in ICH Q5A (R2) guideline “Viral Safety Evaluation of Biotechnology Products Derived from Cell Lines of Human or Animal Origin” (ICH, 2023) but viral contamination from raw materials, etc. should be prevented in a strict manner.

#### 4.3.4 Formulation

If the final product is a cocktail composed of multiple active substances, these substances are generally mixed during the formulation process (Figure 2). For manufacture of cocktail products, multiple phages are individually manufactured using the appropriate phage seed and cell bank and are mixed during the formulation process. In some cocktail products, the phages may interfere with each other. In addition to activity assays for each phage, activity assays for the cocktail product should also be performed.

For the formulation, excipients such as stabilizers are added where necessary. Depending on the type of phages and storage conditions, the product may have a stability problem. In such case, a focus of the examination should be placed on the composition and formulation to improve the stability (Malik et al., 2017).

### 4.4 Specifications and test methods

For PTMPs, which is a drug using viruses as the active ingredients, the quality control is essentially similar to that for live attenuated viral vaccines, recombinant protein products derived from viral vectors, and other viral vector products.

As a part of the control strategy, test items to be performed on the active substance should be identified, and the test methods and acceptance criteria should be established by referring to the ICH Q6B guideline “Specifications: Test Procedures and Acceptance Criteria for Biotechnological/Biological Products” (ICH, 1999). For example, the appearance and description, identification, purity (host cell deoxyribonucleic acid [DNA], host cell proteins, exotoxins), potency (plaque assay or other appropriate methods), quantitative analysis (strength), microbiological tests (sterility, endotoxins, microbial limit, etc.), and pyrogens may be specified.

### 4.5 Biological raw materials

Biological raw materials used in manufacture of drugs must conform to the Standards for biological raw materials (Ministry of Health, Labour and Welfare (MHLW) Notification No. 210 dated 20 May 2003) (MHLW, 2018) from the viewpoint of preventing infections derived from microbial pathogens. In principle, use of biological raw materials should be avoided wherever possible. For cell substrates used in manufacture of general drugs, manufacturing methods without using biological raw materials or with their minimum use have been mostly established. However, the bacteria used as cell substrates for PTMPs are deemed different from the general cell substrates and may require additional biological raw materials. If the use of biological raw materials is unavoidable, raw materials that can comply with the Standards for biological raw materials should be secured. The development should raise awareness of securing biological raw materials of appropriate quality from the early stages, with the possibility of obtaining information from raw material suppliers considered.

## 5 Concept of non-clinical evaluation

### 5.1 Biodistribution

Phages replicate and show antimicrobial activity at the site of infection where target bacteria are densely present, however this replicative ability makes prediction of the biodistribution difficult. Despite this, if the target bacteria are not present, phage are generally rapidly degraded and eliminated.

Treatment of infection with PTMPs requires the phages to be delivered to the infection site where the target bacteria are present. According to a report (Dąbrowska, 2019), however, phages are poorly distributed to tissues, and the antimicrobial activity in the body depends on the route of administration. The route of administration and dosage should be investigated and optimization. Actions for the investigation may include administration of the adequate amount of phages to ensure delivery to the infection site and direct administration into the infection site.

### 5.2 Efficacy evaluation

At present, no procedures for *in vitro* pharmacology studies to evaluate the antimicrobial activity of phages are specified, but potentially applicable evaluation methods are as follows.

- Method to determine phage concentrations at which plaque formation is no longer observed by applying a dilution series of phage suspensions to bacteria spread on the agar medium (plaque method).
- Method to assess whether phages have an antimicrobial activity and replication potential eligible for treatment by determining turbidity of the bacteria culture in a liquid medium after addition of phages (Killing assay).

However, because the evaluation methodology has not been standardized, including the host range, strains, and bacterial volume, its appropriateness should be examined on a case-by-case basis in view of the epidemic status of target pathogens and the expected clinical positioning of the development product.

At the infection site, altered production and environment of enzymes causing degradation and reduced stability of phages may occur in association with the inflammatory reaction. Therefore, it is desirable to evaluate the stability and antimicrobial activity of phages under *in vivo* conditions.

### 5.3 Safety evaluation

In drug development, clinical trials are not started until quality attributes and pharmacological characteristics of the candidate product are appropriately comprehended, and then non-clinical safety evaluation in animals are performed to predict and manage the risks in the first-in-human study and subsequent clinical trials. Human cells do not have receptors necessary for phages to achieve adsorption and infection and thus are not deemed to be targeted by phages (WHO, 2025). Thus, in phage product development, it is required to ensure human safety based on the quality and characteristics of the phage product, within technically feasible and scientifically reasonable limits. Regarding PTMPs, in addition to the potential contamination with unintended pathogenic factors derived from the host bacteria, there are safety concerns related to the spread of virulence genes through transduction and to impurities arising from the manufacturing process. Therefore, it is essential to ensure human safety by minimizing these risks as much as possible. In addition, bacteriolysis of the target bacteria by phages is accompanied by release of bacterial components, including lipopolysaccharide (LPS) and toxins, which may cause events such as inflammatory reactions and shock. On these potential events, appropriate risk minimizing measures should be taken in clinical settings to ensure the safety of the patient.

Non-clinical safety studies or evaluation of PTMPs can be designed by referring to the principles for the non-clinical safety evaluation of monoclonal antibodies and antibody-like proteins that target exogenous molecules (bacteria, viruses, etc.) (ICH, 2011). The U.S. FDA has issued the guideline “Early Clinical Trials with Live Biotherapeutic Products: Chemistry, Manufacturing, and Control (CMC)” on the microbiome (FDA, Center for Biologics Evaluation and Research, 2016), which indicates that the safety should be evaluated in a risk-based manner with the quality control and characterization taken into account from a viewpoint of the safety in humans and also recommends that various toxicity potentials of ingredients in the product are discussed according to the nature of the product and details of a clinical trial to be conducted. The details of non-clinical safety studies to be conducted should be individually determined according to the attributes and development stage of each product. Especially, adequate considerations should be given to conducting non-clinical studies with a focus on the potential risks.

Regarding the immunogenicity, previous studies have suggested that many structural proteins function as immunogens during humoral immune responses to phages. Phage-specific antibodies for a region involved in phage-bacteria interactions could neutralize the phages and attenuate the infectivity of phage to the target strains (Washizaki et al., 2024). It will be useful to evaluate the phage stability in serum, production of phage-specific neutralizing antibodies, cytokine response, and the influence of repeated doses in non-clinical studies (Fujiki et al., 2022; Tan et al., 2024).

## 6 Considerations in clinical trial plan

### 6.1 Clinical evaluation

Treatment of antimicrobial resistant bacterial infections may be clinically evaluated mostly as done with treatment of general infections in terms of evaluation timing and observation items, although it differs in having more varied patient characteristics and posing a higher risk of poor prognosis resulting from treatment failure. Therefore, appendices by disease area in the “Guideline for Clinical Evaluation of Antibacterial Drugs” (dated 23 October 2017 PSEHB/PED Notification No. 1023-3) (Director of Pharmaceutical Evaluation Division, Pharmaceutical Safety and Environmental Health Bureau, MHLW, 2017) can be used as references. In addition, the “Report on Clinical Evaluation of Antimicrobial Agents for AMR” (dated 4 October 2019) (PMDA Subcommittee on AMR of the Science Board, 2019) issued by the Subcommittee on AMR of the Science Board, PMDA and the “Points to Consider for Clinical Development of Drugs Intended for Treatment of Antimicrobial-resistant Gram-negative Bacterial Infections (Early Consideration)” (Administrative Notice dated 24 March 2025) (PMDA Office of New Drug IV, 2025a) may be also referred to.

### 6.2 Route of administration and dose finding

At present, there is no definitive guidance available for the dosage regimen in clinical trials, and thus the dose and route of administration needed to deliver an adequate amount of phages to the target site should be considered based on non-clinical study results. Phages differ from antimicrobial drugs as phages proliferate during infection. To what extent proliferation after administration impacts the dosage regimen warrants future discussion based on the information from clinical and nonclinical studies.

### 6.3 Factors leading to decreased efficacy

Factors potentially leading to post-dose decreased efficacy of a PTMP may include development of the target bacteria’s resistance to the phage and production of phage-specific antibodies.

The target bacteria’s resistance to the phage is often through mutations of the phage receptor molecules in the bacteria (Oechslin, 2018). In addition, some findings have indicated that acquired immune mechanisms such as the CRISPR-Cas system may cause development of resistance to phages. To delay the evolution of bacteria resistant to phages, a phage cocktail composed of multiple phage strains can be used. A report showed that a change in the surface antigen of the bacteria resistant to phages restored the sensitivity to antimicrobial drugs, and another report showed that such change weakened the virulence (Chan et al., 2016; Burmeister et al., 2020; Nakamura et al., 2021).

Some findings showed that phage-specific neutralizing antibodies can affect the serum stability and pharmacokinetics of phages, thereby influencing the clinical outcomes of PTMPs (Fujiki et al., 2022). Given that antibody production is depend on the factors such as route and schedule of phage administration, strategies to mitigate these immune responses could include optimizing the administration route and schedule, as well as use of stabilizers (e.g., hydrogels) as the excipients in formulation process (Washizaki et al., 2024).

In view of the above points, overall evaluation of the efficacy of the development product can effectively leverage data on presence or absence of mutations rendering the bacteria resistant to phages and phage-specific antibodies as well as their impacts obtained from clinical trials.

### 6.4 Concomitant use with antimicrobial drugs

There are *in vitro* reports (Gu Liu et al., 2020; Fujiki et al., 2024; Łusiak-Szelachowska et al., 2022) suggesting that concomitant use of phages with antimicrobial drugs delayed development of resistance to both agents and showing that the concomitant use improved therapeutic outcomes. Whether concomitant use of phages with antimicrobial drugs has an additive or synergistic effect on the efficacy and safety should be investigated after evaluation of the PTMPs. However, as some antimicrobial drugs may inhibit phage proliferation, attention should be paid to selection of the combinations.

## 7 The Cartagena Act

If genetically modified bacteria are used for manufacture or if genetically modified phages are the active ingredients, they are subject to the Cartagena Act (PMDA, 2025c; Sakurai et al., 2023). The Minister of Health, Labour and Welfare is the competent minister in the field of pharmaceutical products, for use of living modified organisms in closed manufacturing facilities, ministerial confirmation of Containment Measures on Type-2 Use in the Cartagena Act is required, and for use of living modified organisms in open environment (including clinical trials and clinical use) as the active ingredients, an approval of Regulations on Type-1 Use in the Cartagena Act is required.

The Containment Measures on Type-2 Use should include appropriate containment measures (physical containment, etc.) according to attributes of the living modified organisms, but for the manufacturing process of phages, necessary containment measures are likely to be similar to ones in BSL facilities required for the bacterial host.

The Regulations on Type-1 Use should include a method of use that is designed to have no impact on the environment even in the case of use in an open system based on attributes of the living modified organisms. The assessment of environmental impacts should pay serious attention to not only attributes of the phage but also the route of administration of the PTMP. The attributes of the phase and the route of administration should be comprehensively evaluated in terms of a range of the impact of its release to the natural environment based on the infectious host range and a possibility of excretion and diffusion of the phage from the route of administration into the natural environment, respectively. In addition, a method of use that prevents or mitigates the potential inadvertent diffusion should be considered depending on the situation.

A phage training result achieved only by subculture may be considered as a spontaneous consequence (natural occurrence). In addition, a gene-deficient phage or bacterial strain generated using recombinant DNA technology may also be considered a consequence of natural occurrences or self-cloning primarily based on the genome sequence. Modified organisms considered as consequences of natural occurrence or self-cloning are not subject to the Cartagena Act (Order of the Ministry of Finance, The Ministry of Education, 2022). Gene-deficient phages or bacterial strains generated using genome editing technology may not be considered as living modified organisms based on the genome sequence, but submission of the information to regulatory authorities is required even if they are not subject to the Cartagena Act (Director of the Pharmaceutical Safety and Environmental Health Bureau, MHLW, 2020). In any case, a method of use that has no adverse impacts on environment should be sought in view of the phage’s ability to kill the target bacteria.

Note that at the research and development phase, the Minister of Education, Culture, Sports, Science and Technology is the competent minister, and containment measures must obey The Ministerial Ordinance Providing Containment Measures to Be Taken in Type 2 Use of Living Modified Organisms for Research and Development (Ministry of Education, Culture, Sports, Science and Technology and the Ministry of the Environment, 2025).

## 8 Closing

PTMPs are increasingly expected to serve as new commercial treatment options alternative to antimicrobial drugs. In Japan, the clinical experience remains limited compared with that in Europe and the U.S. Although pilot studies on phage therapy are underway at the Japan Institute for Health Security (Hayakawa et al., 2025), no clinical trial notifications intended to obtain the regulatory approval have been submitted as of July 2025. Considerations summarized here are expected to encourage development of PTMPs in Japan. Although the discussion here focused on antimicrobial resistant bacterial infections, PTMPs are expected to also be viable for bacterial infections refractory to existing antimicrobial drugs such as biofilm-associated infections and *Clostridioides difficile* infections (Cui et al., 2024; King, 2024). The basic concepts presented here are intended to be applied to antimicrobial resistant bacterial infections targeted by PTMPs but are also applicable to bacterial infections in general.

For individual medicinal product development, utilization of clinical trial consultations (including the consultations for the Cartagena Act) provided by PMDA is encouraged (PMDA, 2025b).
