# Supplementary material for: Regulatory considerations for developing phage therapy medicinal products for the treatment of antimicrobial resistant bacterial infections
Source: Front Pharmacol. 2025 Dec 18;16:1713471. doi: 10.3389/fphar.2025.1713471 (PMC12756395; doi:10.3389/fphar.2025.1713471)
Supplement: Supplementary file 1 [file Table1.docx]

**Supplementary Table 1.** **Clinical trials of phage therapy medicinal products registered in ClinicalTrials.gov** Clinical trials reported to ClinicalTrials.gov since 2020 are listed excluding that have been stopped early or entered a new phase. Information is up to date since the last check on August 31, 2025. Adm. route: Route of administration; IH: Inhalation; IA: Intra-articular injection; IV: Intravenous; IU: Intraurethral/intravesical; NS: Non-specified; NA: Not applicable and est.: estimated. *P. aeruginosa: Pseudomonas aeruginosa; S. aureus: Staphylococcus aureus; E. coli: Escherichia coli; FQR E. coli: fluoroquinolone-resistant E. coli; K. pneumoniae: Klebsiella pneumoniae.*

| Name | Phase | Adm. route | Target | Indication | Start Date | Completion Date | Sponsor or  industrial collaborator | Country | NCT Number |
| --- | --- | --- | --- | --- | --- | --- | --- | --- | --- |
| NS | 3 | NS | Multiple common bacteria | Infective endocarditis | 2025/02/05 | - | University Clinical Hospital na V.V.Vinogradov | Russian Federation | NCT06870409 |
| Preforpro^®^ | 3 | Oral | NS | Vaginal infection | 2024/05/01 (est.) | - | Deerland Enzymes | United  Kingdom | NCT05590195 |
| Pyobacteriophage | 3 | IH | Multiple common bacteria | Acute tonsillitis | 2020/10/02 | - | Tashkent State Medical University (Tashkent Pediatric Medical Institute) | Uzbekistan | NCT04682964 |
| BX004 | 2 | IH | *P. aeruginosa* | Chronic pulmonary infection in cystic fibrosis | 2025/07/02 | - | BiomX, Ltd | United States | NCT06998043 |
| PP1493 & PP1815 | 2 | IA | *S. aureus* | Prosthetic joint infection of the hip or knee | 2025/01/01 (est.) | - | Phaxiam Therapeutics | NS | NCT06605651 |
| TP-102 | 2 | Topical | *P. aeruginosa, S. aureus,* or *Acinetobacter baumannii* | Diabetic foot infection | 2023/11/08 | - | Technophage, SA | United States, India | NCT05948592 |
| AP-PA02 | 2 | IH | *P. aeruginosa* | Chronic pulmonary infection | 2023/01/10 | 2024/08/08 | Armata  Pharmaceuticals, Inc. | United States | NCT05616221 |
| LBP-EC01 | 2 | IU & IV | *E. coli* | Urinary tract infection | 2022/07/13 | - | Locus Biosciences | United States | NCT05488340 |
| NS | 1/2 | IV & IA | *Enterococcus faecium* | Prosthetic joint infection of the hip | 2025/05/01 (est.) | - | Cytophage Technologies Inc. | Canada | NCT06942624 |
| SNIPR001 | 1/2 | Oral | *E. coli* | Hematologic malignancy, scheduled for transplantation harboring FQR *E. Coli* | 2025/02/25 | - | SNIPR Biome Aps. | United States | NCT06938867 |
| NS | 1/2 | IV & IA | *Morganella morganii* | Prosthetic joint infection of the hip | 2025/03/26 | - | Qeen Biotechnologies | Canada | NCT06814756 |
| NS | 1/2 | IV & IA | *S. aureus* | Prosthetic joint infection of the hip | 2024/11/20 | - | Precisio Biotix Therapeutics, Inc. | Canada | NCT06456424 |
| NS | 1/2 | IV | *E. coli* | Urinary tract infection | 2025/06/15 | - | University of California, San Diego | United States | NCT06409819 |
| TP-122A | 1/2 | IH | *P. aeruginosa* | Ventilator-associated pneumonia | 2024/09/01 (est.) | - | Technophage, SA | France | NCT06370598 |
| DUOFAG^®^ | 1/2 | Topical | *S. aureus, P. aeruginosa* | Surgical site infections | 2023/10/27 | - | MB PHARMA s.r.o. | Czechia | NCT06319235 |
| VRELysin™ | 1/2 | Oral | *Enterococcus* | Intestinal infection | 2023/10/25 | - | Intralytix | United States | NCT05715619 |
| NS | 1/2 | Oral, topical and IU | NS | Urinary tract infection | 2023/05/01 | - | Unity Health Toronto | Canada | NCT05537519 |
| WRAIR-PAM-CF1 | 1/2 | IV | *P. aeruginosa* | Infection in cystic fibrosis | 2022/10/03 | 2025/04/10 | National Institute of  Allergy and Infectious Diseases | United States | NCT05453578 |
| AP-SA02 | 1/2 | IV | *S. aureus* | Bacteremia | 2022/04/26 | 2025/01/14 | Armata  Pharmaceuticals, Inc. | United States | NCT05184764 |
| ShigActive™ | 1/2 | Oral | *Shigellosis* | Experimental Shigella challenge | 2023/02/23 | - | Intralytix, Inc. | United States | NCT05182749 |
| BACTELIDE | 1/2 | Topical | *S. aureus, P. aeruginosa,* or *K. pneumoniae* | Pressure ulcer infection | 2022/01/01 (est.) | - | Precisio Biotix Therapeutics, Inc. | United States | NCT04815798 |
| YPT-01 | 1/2 | IH | *P. aeruginosa* | Chronic airway infection in cystic fibrosis | 2021/03/29 | 2023/06/22 | Yale University | United States | NCT04684641 |
| AP-PA02 | 1/2 | IH | *P. aeruginosa* | Chronic pulmonary infection in cystic fibrosis | 2020/12/22 | 2022/12/14 | Armata  Pharmaceuticals, Inc. | United States | NCT04596319 |
| EcoActive | 1/2 | Oral | *E. coli* | Crohn’s Diseases | 2019/05/01 | - | Intralytix, Inc. | United States | NCT03808103 |
| NS | 1/2 | Topical | *S. aureus* | Diabetic foot ulcers infection | 2022/06/01 (est.) | - | Phaxiam Therapeutics | France | NCT02664740 |
| NS | 1 | IV | Multiple common bacteria | Infection in cystic fibrosis | 2025/10/01 (est.) | - | University of California, San Diego | NS | NCT07048704 |
| NS | 1 | IV & IA | *Staphylococcus epidermidis* | Chronic periprosthetic joint infection | 2024/02/22 | - | Cytophage Technologies Inc. | Canada | NCT06827041 |
| NTR-101 | 1 | Oral | *Enterococcus faecalis* | Alcohol-associated hepatitis | 2025/11/05 (est.) | - | Nterica Bio inc | United States | NCT06750588 |
| TAILФR Phage Cocktail | 1 | IU | *E. coli* | Bacteriuria in spinal cord injury | 2025/02/03 | - | Baylor College of Medicine | United States | NCT06559618 |
| HY-133 | 1 | IH | *S. aureus* | NA—Healthy individual | 2024/07/10 | - | University Hospital Tuebingen | Germany | NCT06290557 |
| PGX-0100 | 1 | IH | *S. aureus, P. aeruginosa,* or *K. pneumoniae* | Burn infection | 2022/01/01 (est.) | - | Precisio Biotix Therapeutics, Inc. | Australia | NCT04323475 |
| BX002-A | 1 | Oral | NA | NA—Healthy individual | 2020/10/28 | 2020/12/21 | BiomX, Inc. | United States | NCT04737876 |
| PrePhage | 1 | Nasal | NS | Necrotizing enterocolitis in Preterm infant | 2023/11/07 | - | Rigshospitalet Hospital | Denmark | NCT05272579 |
